# Dietary canolol protects the heart against the deleterious effects induced by the association of rapeseed oil, vitamin E and coenzyme Q10 in the context of a high-fat diet

**DOI:** 10.1186/s12986-018-0252-4

**Published:** 2018-02-13

**Authors:** Thibault Leger, Isabelle Hininger-Favier, Frédéric Capel, Alain Geloen, Jean-Paul Rigaudière, Chrystèle Jouve, Elodie Pitois, Gaelle Pineau, Carole Vaysse, Jean-Michel Chardigny, Marie-Caroline Michalski, Corinne Malpuech-Brugère, Luc Demaison

**Affiliations:** 1 Université Clermont Auvergne, INRA, UNH, Unité de Nutrition Humaine, CRNH Auvergne, 58 rue Montalembert, BP 321, 63009 Clermont-Ferrand cedex 1, France; 2 Univ. Grenoble Alpes, INSERM, LBFA, 38000 Grenoble, France; 3 Univ-Lyon, laboratoire CarMeN, INRA UMR1397, INSERM U1060, Université Claude Bernard Lyon 1, INSA-Lyon, IMBL, 69621 Villeurbanne, France; 4 ITERG-ENMS, Université de Bordeaux, rue Léo Saignat, 33076 Bordeaux cedex, France; 5 Present address: Centre de Recherche INRA Bourgogne Franche Comté, Bâtiment Le Magnen, 17 rue Sully, BP 86510, 21065 Dijon cedex, France

**Keywords:** Heart, Rapeseed oil, ω3 PUFAs, Antioxidant, Canolol, Obesity

## Abstract

**Background:**

Obesity progressively leads to cardiac failure. Omega-3 polyunsaturated fatty acids (PUFA) have been shown to have cardio-protective effects in numerous pathological situations. It is not known whether rapeseed oil, which contains α-linolenic acid (ALA), has a similar protective effect. Omega-3 PUFAs are sensitive to attack by reactive oxygen species (ROS), and lipid peroxidation products could damage cardiac cells. We thus tested whether dietary refined rapeseed oil (RSO) associated with or without different antioxidants (vitamin E, coenzyme Q10 and canolol) is cardio-protective in a situation of abdominal obesity.

**Methods:**

Sixty male Wistar rats were subdivided into 5 groups. Each group was fed a specific diet for 11 weeks: a low-fat diet (3% of lipids, C diet) with compositionally-balanced PUFAs; a high-fat diet rich in palm oil (30% of lipids, PS diet); the PS diet in which 40% of lipids were replaced by RSO (R diet); the R diet supplemented with coenzyme Q10 (CoQ10) and vitamin E (RTC diet); and the RTC diet supplemented with canolol (RTCC diet). At the end of the diet period, the rats were sacrificed and the heart was collected and immediately frozen. Fatty acid composition of cardiac phospholipids was then determined. Several features of cardiac function (fibrosis, inflammation, oxidative stress, apoptosis, metabolism, mitochondrial biogenesis) were also estimated.

**Results:**

Abdominal obesity reduced cardiac oxidative stress and apoptosis rate by increasing the proportion of arachidonic acid (AA) in membrane phospholipids. Dietary RSO had the same effect, though it normalized the proportion of AA. Adding vitamin E and CoQ10 in the RSO-rich high fat diet had a deleterious effect, increasing fibrosis by increasing angiotensin-2 receptor-1b (Ag2R-1b) mRNA expression. Overexpression of these receptors triggers coronary vasoconstriction, which probably induced ischemia. Canolol supplementation counteracted this deleterious effect by reducing coronary vasoconstriction.

**Conclusion:**

Canolol was found to counteract the fibrotic effects of vitamin E + CoQ10 on cardiac fibrosis in the context of a high-fat diet enriched with RSO. This effect occurred through a restoration of cardiac Ag2R-1b mRNA expression and decreased ischemia.

## Background

The prevalence of obesity in the Western world has increased dramatically in the past two decades [1]*.* Obesity, and especially central or abdominal obesity, is strongly associated with the occurrence of metabolic syndrome and type-2 diabetes mellitus which increase the risk of developing cardiovascular disease. Insulin resistance and hyperglycemia are associated with oxidative stress and inflammation [2] which affect the vessels and induce atherosclerosis [3, 4]. Moreover, obesity also alters cardiac function, leading to heart failure in the long term. A preliminary step that increases cardiac mechanical function leads to increased proportion of arachidonic acid (C20:4ω6 or AA) in the cardiac membrane and increased coronary microvessel vasodilatation capacities [5]. However, this status is transient: heart failure inexorably develops [6] due to the progressive development of post-prandial hyperglycemia and oxidative stress. These changes alter coronary microvessel function [7], reduce myocardial perfusion and decrease cardiac mechanical function.

Partial substitution of ω6 PUFA by ω3 PUFA such as α-linolenic acid (C18:3ω3 or ALA), eicosapentaenoic acid (C20:5ω3 or EPA) and docosahexaenoic acid (C22:6ω3 or DHA) in the diet is known to reduce the proportion of AA in cardiac phospholipids [8]. It can thus prevent obesity-induced hyper-activation of cardiac function and slow the development of cardiac insufficiency. These fatty acids also act on systemic glucose metabolism. It was recently shown that dietary EPA reduces high-fat diet-induced insulin resistance [9], and may thus help slow progression toward heart failure.

Dietary ω3 PUFA have long been shown to protect the heart against several diseases such as atherosclerosis [10], ischemia/reperfusion [11] and hypertrophy [12]. However, ω3 PUFA are also very vulnerable to ROS attack, much more so than ω6 PUFA. ROS attack can occur in the diet through food oxygenation and intake. ω3 PUFA are often protected by an adequate type and amount of antioxidants (vitamin E, rosmarinic acid, etc.), but their dispersion in the whole organism including biological membranes necessitates specific anti-oxidative properties. Indeed, the body can play host to strong ROS production, particularly during pathophysiological conditions. The mitochondria, but also several enzymes such as the NADPH oxidase and xanthine oxidase, are a source of ROS. The low amount of antioxidants associated with dietary ω3 PUFA may not be high enough to protect the ω3 PUFA dispersed in the whole organism. Furthermore, the type of antioxidants may be ill-suited to converge toward the intracellular site of ROS production. It thus appears logical to treat progressive obesity with an adequate amount of appropriate antioxidants in order to protect the ω3 PUFA in the whole organism when oxidative stress is high.

Rapeseed oil contains approximately 8–10% ALA and is a common dietary source of lipids in human nutrition. Rapeseed oil also contains several antioxidants, namely vitamin E, coenzyme Q10 (CoQ10) and phenolic compounds such as canolol, sinapic acid and sinapin. ALA has well-document cardio-protective effects [13–16], namely for the prevention of coronary heart disease, sudden cardiac death, non-fatal acute myocardial infarction, heart failure and stroke, but apart from CoQ10 against heart failure, there is still controversy over whether the other antioxidants such as vitamin E have beneficial effects [17].

Here we report a study designed to evaluate the effects of ALA-rich rapeseed oil on key aspects of cardiac function (fibrosis, inflammation, oxidative stress, apoptosis, metabolism, mitochondrial biogenesis) in male Wistar rats in the context of a high-fat diet, and to test whether the addition of different rapeseed-sourced antioxidants (vitamin E, CoQ10 and canolol) improved the effects of refined rapeseed oil.

## Methods

### Oil preparation

Refined palm oil was furnished by the Société Industrielle des Oléagineux (Saint-Laurent-Blangy, France). Refined rapeseed and sunflower oils were prepared by the French Institute for Fats and Oils Research (ITERG, Pessac, France). Canolol was prepared by the ITERG through thermal treatment of rapeseed crops. Alpha-tocopherol was purchased from Sigma (Saint-Quentin-Fallavier, France). CoQ10 was kindly gifted by Kaneka Nutrients (Pasadena, TX). Two fortified rapeseed oil mixtures were prepared: the first one was enriched with 1700 mg/kg α-tocopherol and 300 mg/kg CoQ10 (RTC) and the second one had the same composition but also contained canolol as 600 sinapic acid-equivalents (RTCC). Details of the exact composition of the different lipid preparations are reported in Table 1.

**Table 1:** Micronutrient and fatty acid compositions of the different lipid fractions

	PS	R	RTC	RTCC
Palm oil	90	60	60	60
Sunflower oil	10			
Rapeseed oil	-	40	40	40
α-toco (mg/kg)	125	180	2140	2020
CoQ10 (mg/kg)	20	20	260	260
Canolol (eq. S)	–	–	–	600
FA composition				
C16:0	40	28.3	28.4	28.6
C18:0	4.2	3.2	3.2	3.2
SFA	46.3	33.2	33.3	33.5
C18:1	43.5	48.7	48.8	48.6
MUFA	43.9	49.7	49.7	49.5
C18:2ω6	9	13.2	13.1	13.1
C18:3ω3	0.2	3.1	3.1	3.1
Trans FA	0.6	0.8	0.8	0.7

*PS* palm oil/sunflower oil mixture, *R* rapeseed oil, *RTC* α-tocopherol + coenzyme Q10 mixture in rapeseed oil, *RTCC* same mixture as RTC + canolol, *α-toco* α-tocopherol, *CoQ10* coenzyme Q10, *eq. S* sinapic acid equivalent, *FA* fatty acid, *SFA* saturated fatty acid, *MUFA* monounsaturated fatty acid

### Animals and diets

Sixty male Wistar rats weighing 220–250 g were purchased from Janvier SA (Le Genest Saint-Isle, France) then housed 4 per cage in an animal facility controlled for temperature (22 °C) and light/dark cycles (12 h/12 h). After 2 weeks of chow diet, they were divided into 5 groups of 12 animals. Each group was nourished with a specific diet for 11 weeks: a low-fat diet (3% of lipids, C diet) with compositionally-balanced PUFAs; a high-fat diet rich in palm oil (30% of lipids, PS diet); the PS diet in which 40% of lipids were replaced by RSO (R diet); the R diet supplemented with coenzyme Q10 (CoQ10) and vitamin E (RTC diet); and the RTC diet supplemented with canolol (RTCC diet). The lipid composition of these diets is presented in Table 1. At the end of the feeding period, the animals were sacrificed under anesthesia and their hearts were rapidly collected and frozen in liquid nitrogen. The samples were pulverized in liquid nitrogen and the powder was stored at − 80 °C until the biochemical determinations were performed.

### Western blotting

Tissues were ground three times in a mini-bead beater (Minilys System, Ozyme, Saint Quentin en Yvelines, France) in presence of a lysis buffer constituted of HEPES 50 mM, sodium chloride 150 mM, EDTA 10 mM, anhydrous sodium tetrabasic pyrophosphate 10 mM, β-glycerophosphate 25 mM, sodium fluoride 100 mM and anhydrous glycerol 1.086 M supplemented with phosphatase inhibitors (Sigma Aldrich, Saint-Quentin-Fallavier, France). Successive centrifugations were performed in order to collect the supernatants. Protein quantifications were performed using a bicinchoninic acid assay kit (Thermo Scientific, Rockford, IL). For protein immunoblotting, 20 μg of proteins were loaded for separation by SDS-PAGE electrophoresis and transfer on PVDF membranes. Membranes were then immunoblotted with the appropriate antibody to detect glyceraldehyde 3-phosphate dehydrogenase (GAPDH), serine 473 phosphorylated Akt, total Akt, cleaved caspase 3, transforming growth factor-β1 (TGF-β1), matrix metallopeptidase-9 (MMP9) and nuclear factor of kappa light polypeptide gene enhancer in B-cells inhibitor, alpha (IκBα). Antibody binding was detected using HRP-conjugated secondary antibodies and ECL western blotting substrate (Thermo Scientific, Rockford, IL). Immunoblots were visualized via a chemoluminescence imaging system (MF ChemiBIS, DNR bio imaging systems, Jerusalem, Israel) and quantified using MultiGauge V3.2 software.

### Gene expression analysis

Total RNA were extracted from 50 mg of cardiac powder using TRIzol® (Thermo Scientific, Rockford, IL) according to the manufacturer’s instructions. RNA quantification and integrity were verified by measuring the ratio of optical density at 260 and 280 nm and by agarose gel, respectively. cDNA was synthesized from 2 μg of total RNA using a High-Capacity cDNA Reverse Transcription Kit from Applied Biosystems (Thermo Scientific, Rockford, IL). The reverse transcription products were used for quantitative real-time polymerase chain reaction (qRT-PCR) using specific primers and Rotor-Gene SYBR Green PCR master mix on a Rotor-Gene Q system (Qiagen, Courtaboeuf, France). Messenger RNA (mRNA) was quantified using the standard curve of native cDNA and serial dilutions. mRNA expressions were determined for p53, PGC1-α, angiotensin-2, angiotensin-2 receptors-1a and -1b, pyruvate dehydrogenase 4, superoxide dismutase 2, glutathione peroxidase 4, catalase, ICAM-1, VCAM-1 and nitric oxide synthase 3. Primer sequences and PCR conditions can be made available on request (luc.demaison@inra.fr). β-actin and non-POU domain-containing octamer-binding protein (NoNo) genes were used as housekeeping genes.

### Fatty acid analysis

Fatty acid profiling of cardiac phospholipids was performed by gas chromatography–flame ionization detection (GC-FID). Briefly, total lipids were extracted from cardiac tissues as per Floch et al. [18] and the organic phase was evaporated under nitrogen. Phospholipids were separated from non-phosphorus lipids using a Sep-Pak cartridge (Chromabond, Macherey-Nagel, Düren, Germarny) [19]. Fatty acid methyl esters (FAMEs) were prepared via basic trans-esterification followed by acid trans-esterification, and analyzed using a silica CP-Sil 88 capillary column (100 m/0.25 mm internal diameter/0.20 μm film thickness; Varian, Palo Alto, CA) on a GC system (Thermo Electron Corp.; Waltham, MA) equipped with a flame ionization detector.

### Cardiac oxidative stress

Protein oxidation in the heart was evaluated by the disappearance of protein thiol groups [20]. Thiols were assayed using 5,5′-dithiobis(2-nitrobenzoic acid (DTNB)) to derive the thiol groups. The calibration curve was obtained by mixing two stock solutions of N-acetyl cystein (NAC) in the range of 0.125–0.6 mmol/L. Samples were measured spectrophotometrically at 415 nm (Hitachi 912, B Braun Science Tec, France) in the presence of a phosphate buffer 50 mM, EDTA 100 mM, pH 8 and bis-5,5′-dithio-bis(2-nitrobenzoic acid) 10 mM.

Thiobarbituric acid reactive substances (TBARS) were assayed as per Poubelle et al. [21].

The antioxidant status of the heart was evaluated using the ferric reducing antioxidant power (FRAP) assay as a global marker of antioxidant power. The FRAP assay uses antioxidants as reductants in a redox-linked colorimetric method. In this assay, at low pH, a ferric-tripyridyltriazine (Fe^III^-TPTZ) complex is reduced to the ferrous form, which is blue and monitored by measuring the change in absorption at 593 nm. The change is directly proportional to the reducing power of the electron-donating antioxidants present in the tissue. The absorbance change is translated into a FRAP value (in μmol/L) by relating the change of absorbance at 593 nm of test sample to that of a standard solution of known FRAP value.

Glutathione peroxidase (GPx) activity, measuring a seleno-enzyme involved in protection against H_2_O_2_, was evaluated by the modified method of Flohé & Gunzler [22] using tert-butyl hydroperoxide (Sigma Chemical Co, Paris, France) as substrate instead of hydrogen peroxide.

SOD was assayed using a commercially-available kit (Sigma Aldrich, Saint-Quentin-Fallavier, France).

### Other biochemical determinations

Myocardial caspase 3 activity and collagen content were evaluated using commercially available kits from Abcam (Paris, France).

Cardiac lipids (triglycerides, diglycerides, free fatty acids, cholesterol, cholesterol esters and phospholipids) were determined by flame ionization detection by the Iatroscan method [23].

### Statistical analysis

Results are presented as means ± S.E.M. Data were tested by one-way analysis of variance (ANOVA) performing the following comparisons: PS vs. C, R vs. PS, RTC vs. R, RTCC vs. RTC. Group means were compared with a Fisher’s LSD test. A probability (p) less than 0.05 was considered significant. All statistical analysis was performed using NCSS 2007 software.

## Results

### General data

The high-fat diets tended to increase animal weight (477 ± 14, 469 ± 13, 460 ± 12 and 459 ± 11 g for the PS, R, RTC and RTCC groups, respectively) compared to the control diet (435 ± 7), but the differences were not significant. However, the high-fat diets strongly increased abdominal adiposity (by 36 ± 2, 37 ± 3, 34 ± 2 and 36 ± 2 g for the PS, R, RTC and RTCC groups vs. 21 ± 1 g for the C group), but not enough to increase animal weight. The heart weight was similar in all the dietary groups (209 ± 7, 219 ± 8, 211 ± 9, 211 ± 8 and 222 ± 8 mg of dry weight for the C, PS, R, RTC and RTCC groups). Similarly, the heart weight to body weight ratios were unaffected by the dietary manipulations (data not shown).

### Fatty acid composition of cardiac phospholipids

The most significant results presented in Table 2 are the followings: i) the PS diet strongly increased the proportion of AA (+ 20%, *p* < 0.001), C22:4ω6 (+ 20%, *p* < 0.01) and C22:5ω6 (+ 377%, *p* < 0.001) compared to the control group. This was due to a decrease in linoleic acid (C18:2ω6 or LA, − 49%, *p* < 0.001). This diet reduced the proportions of individual ω3 fatty acids (− 87, − 25, − 53 and − 35% for the ALA, EPA, C22:5ω3 and DHA, *p* < 0.001, 0.05, 0.001 and 0.001, respectively). It also decreased the PUFA/SFA ratio (− 17%, *p* < 0.001); ii) the rapeseed oil-rich diets decreased the proportions of AA (− 18% in general, *p* < 0.001), C22:4ω6 (− 59% in general, *p* < 0.001) and C22:5ω6 (− 93% in general, *p* < 0.001) compared to the PS diet, and slightly increased that of LA (+ 30% in general, *p* < 0.001). They also raised the proportions of ALA (+ 915% in general, *p* < 0.001), docosapentaenoic acid (C22:5ω3 or DPA, + 368% in general, *p* < 0.001) and DHA (+ 214% in general, *p* < 0.001). PUFA/SFA ratio was slightly but significantly increased by rapeseed oil-rich diets (+ 5, + 8 and + 7%, *p* < 0.05, 0.001 and 0.01, for the R, RTC and RTCC groups compared to the PS group); iii) the diet combining α-tocopherol, CoQ10 and rapeseed oil and the same diet with added polyphenol canolol had little effect on cardiac phospholipid fatty acid composition compared to the R diet.

**Table 2:** Fatty acid composition of cardiac phospholipids

	C	PS	R	RTC	RTCC
12:0	0.03 ± 0.01	0.04 ± 0.01	0.03 ± 0.01	0.03 ± 0.01	0.03 ± 0.01
14:0	0.17 ± 0.01^a^	0.15 ± 0.01^ac^	0.13 ± 0.01^cd^	0.12 ± 0.01^bde^	0.13 ± 0.01^ce^
15:0	0.09 ± 0.01^a^	0.03 ± 0.01^b^	0.04 ± 0.01^b^	0.04 ± 0.01^b^	0.04 ± 0.01^b^
DMA 16:0	2.33 ± 0.06^a^	3.14 ± 0.05^b^	2.74 ± 0.14^c^	2.88 ± 0.06^c^	2.85 ± 0.06^c^
16:0	11.90 ± 0.33	11.98 ± 0.29	12.11 ± 0.34	11.85 ± 0.16	12.35 ± 0.28
17:0	0.32 ± 0.01^a^	0.12 ± 0.01^b^	0.14 ± 0.01^b^	0.13 ± 0.01^b^	0.14 ± 0.01^b^
DMA 18:0	0.81 ± 0.09^a^	1.02 ± 0.06^b^	0.91 ± 0.03^ab^	1.00 ± 0.09^ab^	0.93 ± 0.05^ab^
18:0	19.53 ± 0.31^a^	22.25 ± 0.29^b^	21.27 ± 0.48^c^	21.17 ± 0.09^c^	20.88 ± 0.07^c^
20:0	0.18 ± 0.02^ac^	0.17 ± 0.02^a^	0.23 ± 0.01^bd^	0.25 ± 0.01^bd^	0.22 ± 0.01^cd^
22:0	0.16 ± 0.01^a^	0.31 ± 0.01^b^	0.21 ± 0.01^c^	0.22 ± 0.02^c^	0.20 ± 0.01^c^
24:0	0.01 ± 0.01^a^	0.08 ± 0.01^b^	0.02 ± 0.01^a^	0.02 ± 0.01^a^	0.02 ± 0.01^a^
SFA	35.21 ± 0.33^a^	39.29 ± 0.24^b^	37.86 ± 0.50^c^	37.56 ± 0.21^c^	37.56 ± 0.16^c^
16:1ω9	0.10 ± 0.01^a^	0.08 ± 0.01^b^	0.10 ± 0.01^a^	0.08 ± 0.01^bc^	0.09 ± 0.01^ac^
16:1ω7	0.47 ± 0.05^a^	0.1 ± 0.01^b^	0.08 ± 0.02^b^	0.08 ± 0.01^b^	0.11 ± 0.02^b^
18:1ω9	3.89 ± 0.22^a^	6.79 ± 0.45^b^	7.21 ± 0.60^b^	6.09 ± 0.20^b^	6.60 ± 0.40^b^
18:1ω7	4.09 ± 0.10^a^	2.50 ± 0.02^b^	2.88 ± 0.04^c^	2.93 ± 0.05^c^	3.00 ± 0.04^c^
20:1ω9	0.11 ± 0.01^a^	0.06 ± 0.01^b^	0.06 ± 0.01^b^	0.05 ± 0.01^b^	0.05 ± 0.01^b^
MUFA	8.66 ± 0.34^a^	9.50 ± 0.47^ab^	10.32 ± 0.62^b^	9.21 ± 0.16^ab^	9.78 ± 0.47^ab^
18:2ω6	24.22 ± 0.87^a^	12.24 ± 0.46^b^	14.28 ± 0.57^c^	15.48 ± 0.56^c^	14.72 ± 0.31^c^
18:3ω6	0.06 ± 0.01^a^	0.07 ± 0.01^a^	0.14 ± 0.01^c^	0.11 ± 0.01^b^	0.12 ± 0.01^bc^
20:2ω6	0.19 ± 0.01^a^	0.10 ± 0.01^b^	0.11 ± 0.01^b^	0.11 ± 0.01^b^	0.12 ± 0.01^b^
20:3ω6	0.31 ± 0.01^a^	0.38 ± 0.01^b^	0.43 ± 0.03^c^	0.50 ± 0.02^d^	0.46 ± 0.02^cd^
20:4ω6	21.70 ± 0.34^a^	26.13 ± 0.50^b^	21.76 ± 0.76^a^	21.52 ± 0.45^a^	20.68 ± 0.39^a^
22:4ω6	1.21 ± 0.05^a^	1.44 ± 0.04^b^	0.61 ± 0.01^c^	0.58 ± 0.02^c^	0.56 ± 0.02^c^
22:5ω6	1.38 ± 0.09^a^	6.58 ± 0.58^b^	0.48 ± 0.05^c^	0.46 ± 0.02^c^	0.50 ± 0.03^c^
ω6 PUFA	48.73 ± 0.52^a^	46.93 ± 0.53^b^	37.82 ± 0.41^c^	38.77 ± 0.50^c^	37.74 ± 0.35^c^
18:3ω3	0.15 ± 0.01^a^	0.02 ± 0.01^b^	0.22 ± 0.02^c^	0.19 ± 0.01^c^	0.20 ± 0.01^c^
20:5ω3	0.12 ± 0.01^a^	0.09 ± 0.01^b^	0.10 ± 0.01^ab^	0.11 ± 0.01^ab^	0.10 ± 0.01^ab^
22:5ω3	1.22 ± 0.07^a^	0.57 ± 0.02^b^	2.42 ± 0.12^c^	2.71 ± 0.16^cd^	2.88 ± 0.18^d^
22:6ω3	5.59 ± 0.27^a^	3.61 ± 0.15^b^	11.26 ± 0.39^c^	11.47 ± 0.38^c^	11.28 ± 0.36^c^
ω3 PUFA	7.09 ± 0.32^a^	4.28 ± 0.16^b^	14.0 ± 0.40^c^	14.48 ± 0.36^c^	14.45 ± 0.51^c^
PUFA	55.53 ± 0.28^a^	51.21 ± 0.52^cd^	51.82 ± 0.59^bd^	52.98 ± 0.21^b^	52.37 ± 0.54^bc^
ω6/ω3	6.99 ± 0.38^a^	11.09 ± 0.47^b^	2.72 ± 0.08^c^	2.69 ± 0.10^c^	2.60 ± 0.10^c^
EPA + DHA	5.71 ± 0.27^a^	3.70 ± 0.16^b^	11.36 ± 0.39^c^	11.58 ± 0.37^c^	11.37 ± 0.36^c^
EPA/AA (× 10^3^)	5.08 ± 0.44^a^	3.36 ± 0.44^b^	4.61 ± 0.31^a^	5.07 ± 0.43^a^	4.62 ± 0.29^a^
(EPA + DHA)/AA	0.27 ± 0.01^a^	0.14 ± 0.01^b^	0.53 ± 0.02^c^	0.54 ± 0.02^c^	0.55 ± 0.02^c^
PUFA/SFA	1.563 ± 0.005^a^	1.304 ± 0.018^b^	1.371 ± 0.028^c^	1.406 ± 0.012^c^	1.395 ± 0.019^c^

*C* control rats, *PS* rats fed a palm oil/sunflower oil mixture, *R* rats fed rapeseed oil, *RTC* rats fed rapeseed oil enriched with α-tocopherol and coenzyme Q10, *RTCC* rats fed RTC plus canolol, *DMA* dimethylacetal, *SFA* saturated fatty acid, *MUFA* monounsaturated fatty acid, *PUFA* polyunsaturated fatty acid, *EPA* eicosapentaenoic acid or C20:5ω3, *DHA* docosahexaenoic acid or C22:6ω3, *AA* arachidonic acid or C20:4ω6. Averages of 5 rats per group

^a, b, c, d, e^means in a row without a common letter are significantly different

### Myocardial mRNAs for angiotensin 2 and associated receptors

**Fig. 1:**
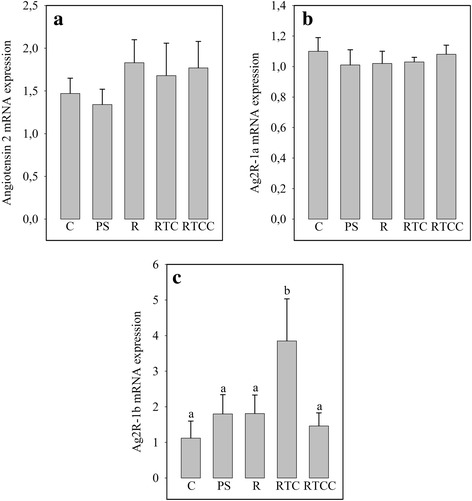
Influence of the different diets on myocardial mRNA levels for angiotensin 2 (panel **a**), angiotensin 2 receptor-1a (Ag2R-1a, panel **b**) and angiotensin 2 receptor-1b (Ag2R-1a, panel **c**). Figures are averages of 12 rats per group. C: rats fed the control diet; PS: rats fed with the high-fat diet rich in saturated and monounsaturated fatty acids; R: rats fed the high-fat diet rich in rapeseed oil; RTC: rats fed with the same diet as R, but enriched with vitamin E and CoQ10; RTCC: rats fed the same diet as RTC, but enriched with canolol; a,b: In a given panel, histograms without a common letter are significantly different

Myocardial angiotensin 2 mRNA expression was unaffected by the different diets (Fig. 1a). Cardiac angiotensin 2 receptor type 1a mRNA expression was also unchanged (Fig. 1b). In contrast, angiotensin 2 receptor type 1b expression was significantly increased by the RTC diet compared with the other four diets (+ 149%, Fig. 1c).

### Myocardial collagen content

**Fig. 2:**
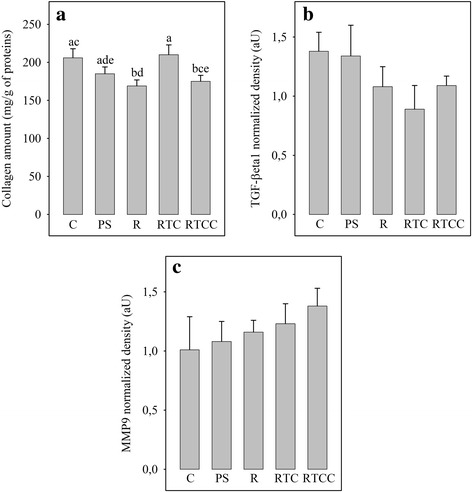
Myocardial contents of collagen (panel **a**), TGF-β1 (panel **b**) and MMP9 (panel **c**). Averages of 12 rats per group. C: rats fed the control diet; PS: rats fed the high-fat diet rich in saturated and monounsaturated fatty acids; R: rats fed with high-fat diet rich in rapeseed oil; RTC: rats fed with the same diet as R, but enriched with vitamin E and CoQ10; RTCC: rats fed the same diet as RTC, but enriched with canolol; TGF-b1: transforming growth factor-β1; MMP9: matrix metallopeptidase 9; GAPDH: glyceraldehyde-3-phosphate dehydrogenase; a,b,c,d,e: In a given panel, histograms without a common letter are significantly different

The myocardial collagen content of the 5 dietary groups is shown in Fig. 2a. High-fat diets led to lower collagen content of myocardial tissue compared to C diet, especially with R and RTCC diets (− 18 and − 15%, *p* < 0.05) but not the RTC diet which maintained a high cardiac collagen content (+ 24% vs R group and + 20% vs RTCC group, *p* < 0.05). The observed changes in collagen content were not associated with significant changes in amount of TGF-β1 (Fig. 2b) and matrix metalloproteinase-9 (Fig. 2c).

### Myocardial metabolism

In terms of metabolism (Table 3), the PS diet reduced myocardial diglyceride content (− 36%, *p* < 0.05) compared to the C group. However, PS diet did not alter the other metabolic parameters determined here (ratio between phosphorylated and non-phosphorylated protein kinase B, pyruvate dehydrogenase kinase-4 mRNA expression, myocardial contents of triglycerides, NEFA, cholesterol, cholesterol esters and phospholipids). Compared to the PS diet, the R diet increased cardiac diglyceride level (+ 44%, *p* < 0.01) but did not change the other parameters. The RTC diet led to a strong increase in pyruvate dehydrogenase kinase-4 mRNA expression compared to the R diet (+ 54%, *p* < 0.05). However, comparisons of RTC vs. R diets did not reveal significant differences in the other metabolic parameters. Finally, compared to RTC diet, the RTCC diet significantly normalized pyruvate dehydrogenase kinase-4 mRNA expression and myocardial triglyceride content (− 46 and − 23%, p < 0.05 and 0.05, respectively) down to the level observed with the R diet.

**Table 3:** Myocardial glucose and lipid metabolism

	C	PS	R	RTC	RTCC
pAkt/totAkt	1.64 ± 0.32	1.52 ± 0.25	1.20 ± 0.29	1.29 ± 0.25	1.52 ± 0.23
PDK4	0.13 ± 0.02^a^	0.28 ± 0.07^ab^	0.41 ± 0.08^b^	0.63 ± 0.09^c^	0.34 ± 0.03^b^
TG	20 ± 1^a^	25 ± 2^ab^	25 ± 2^ab^	31 ± 4^b^	24 ± 2^a^
DG	1.4 ± 0.2^ac^	0.9 ± 0.09^b^	1.3 ± 0.1^cd^	1.1 ± 0.1^bd^	0.9 ± 0.1^b^
NEFA	1.2 ± 0.2^a^	1.1 ± 0.1^ab^	1.1 ± 0.1^ab^	0.9 ± 0.1^b^	0.9 ± 0.1^b^
Chol	3.2 ± 0.3^a^	2.7 ± 0.3^ab^	2.5 ± 0.1^b^	2.4 ± 0.1^b^	2.4 ± 0.1^b^
CE	2.6 ± 0.6	2.3 ± 0.6	1.1 ± 0.2	1.8 ± 0.4	2.0 ± 0.6
PL	70 ± 3	68 ± 2	65 ± 4	63 ± 4	70 ± 3

*C* control rats, *PS* rats fed a palm oil/sunflower oil mixture, *R* rats fed rapeseed oil, *RTC* rats fed rapeseed oil enriched with α-tocopherol and coenzyme Q10, *RTCC* rats fed RTC plus canolol, *pAkt/totAKt* phosphorylated protein kinase B-to-total protein kinase B ratio, *PDK4* pyruvate dehydrogenase kinase-4 mRNA expression, *TG, DG, NEFA, Chol, CE, PL* amounts of triglycerides, diacylglycerols, non-esterified fatty acids, cholesterol, cholesterol esters and phospholipids, respectively, in the myocardium. Averages of 12 rats per group. Lipid amounts are expressed in mg/g of heart weight

^a, b, c, d^means in a row without a common letter are significantly different

### Oxidative stress

Several features of myocardial oxidative stress are presented in Table 4. Compared to the C diet, the PS diet strongly decreased the myocardial content of TBARS (− 21, *p* < 0.01), total antioxidant capacities (FRAP, − 10%, *p* < 0.05) and SOD activity (− 53%, *p* < 0.01) without altering the other parameters of oxidative stress. Adding RSO to the high-fat diet (R diet) increased the amount of thiol groups (+ 12%, *p* < 0.001) compared to the PS diet, suggesting reduced protein oxidative stress. Enrichment of the R diet with vitamin E and CoQ10 (RTC diet) increased GPx activity (+ 9%, *p* < 0.05) and SOD2 (+ 46%, *p* < 0.05) and GPX4 (+ 20%, *p* < 0.05) mRNA expression. Further addition of canolol had little effect other than decreasing SOD2 mRNA expression (− 31%, *p* < 0.05) back down to the level observed with the R diet.

**Table 4:** Oxidative stress

	C	PS	R	RTC	RTCC
Thiols	55 ± 1^a^	57 ± 1^a^	64 ± 1^b^	62 ± 2^b^	65 ± 1^b^
TBARS	0.29 ± 0.01^a^	0.23 ± 0.01^b^	0.23 ± 0.02^b^	0.24 ± 0.02^b^	0.24 ± 0.01^b^
FRAP	107 ± 4^a^	96 ± 3^c^	88 ± 2^cb^	88 ± 3^cb^	86 ± 3^b^
SOD activity	358 ± 55^a^	170 ± 20^b^	167 ± 15^b^	158 ± 11^b^	132 ± 21^b^
GPX activity	1062 ± 22^a^	1065 ± 28^a^	1066 ± 26^a^	1160 ± 25^b^	1096 ± 26^ab^
SOD2	0.41 ± 0.03^a^	0.55 ± 0.09^a^	0.57 ± 0.08^a^	0.83 ± 0.10^b^	0.57 ± 0.08^a^
GPX4	0.54 ± 0.01^a^	0.59 ± 0.02^ab^	0.55 ± 0.03^a^	0.66 ± 0.04^b^	0.64 ± 0.04^b^
Cat	0.30 ± 0.05^a^	0.51 ± 0.11^ab^	0.57 ± 0.09^ab^	0.79 ± 0.15^b^	0.56 ± 0.11^ab^

*C* control rats, *PS* rats fed a palm oil/sunflower oil mixture, *R* rats fed rapeseed oil, *RTC* rats fed rapeseed oil enriched with α-tocopherol and coenzyme Q10, *RTCC* rats fed RTC plus canolol, *TBARS* thiobarbituric acid reactive substances, *FRAP* ferric reducing antioxidant power, *SOD* superoxide dismutase, *GPX* glutathione peroxidase, *SOD2, GPX4 and Cat* SOD2, GPX4 and catalase mRNA expression. Thiols, TBARS and FRAP are expressed in μmoles/g of proteins. SOD and GPX activities are expressed in in U/g of proteins. Averages of 12 rats per group

^a, b, c^means in a row without a common letter are significantly different

### Apoptosis

**Fig. 3:**
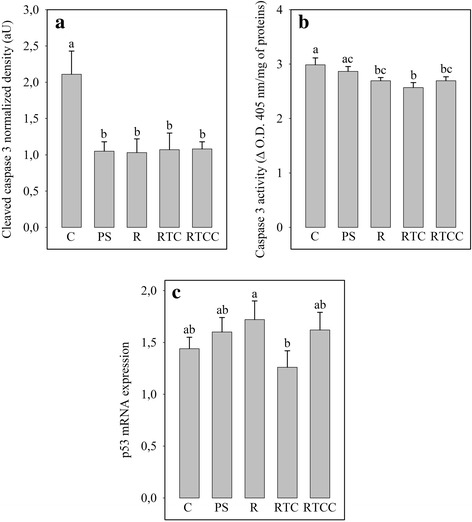
Myocardial apoptosis estimated as the amount of cleaved caspase 3 (panel **a**), caspase 3 activity (panel **b**) and p53 mRNA level (panel **c**). Figures are averages of 12 rats per group. C: rats fed the control diet; PS: rats fed the high-fat diet rich in saturated and monounsaturated fatty acids; R: rats fed the high-fat diet rich in rapeseed oil; RTC: rats fed the same diet as R, but enriched with vitamin E and CoQ10; RTCC: rats fed with the same diet as RTC, but enriched with canolol; Δ O.D.: change in optical density. a,b,c: In a given panel, histograms without a common letter are significantly different

Western blotting analysis (Fig. 3a) indicated that the cardiac level of the cleaved caspase-3 was reduced with the PS diet (− 50%, *p* < 0.05) compared to the C diet, suggesting that apoptosis was less intense in this group. Caspase-3 activity (Fig. 3b) and p53 mRNA expression (Fig. 3c) were not significantly reduced. Adding RSO to the PS diet did not modify the situation. Likewise, adding vitamin E and CoQ10 to the R diet did not alter the severity of apoptosis but did significantly reducee p53 mRNA expression compared to the RTC diet (− 27%, p < 0.05). Finally, adding canolol to the RTC diet did not alter the protein expression of cleaved caspase-3 and caspase-3 activity compared to the R and RTC diets, but tended to bring p53 mRNA expression back down to the level observed with the R diet. The differences in apoptosis intensity were not due to activation of the inflammation pathway (Table 5): neither the amount of IκBα protein nor the mRNA levels for ICAM, VCAM and nitric oxide synthase-3 were altered by the different diets.

**Table 5:** Activation of the inflammation pathway

	C	PS	R	RTC	RTCC
IκBα	1.57 ± 0.13	1.38 ± 0.11	1.31 ± 0.09	1.38 ± 0.06	1.48 ± 0.16
ICAM	1.67 ± 0.47	1.7 ± 0.45	2.01 ± 0.50	1.89 ± 0.48	1.1 ± 0.22
VCAM	0.84 ± 0.22	0.92 ± 0.22	0.87 ± 0.18	1.11 ± 0.27	1.13 ± 0.21
NOS3	1.06 ± 0.25	0.83 ± 0.17	0.89 ± 0.16	0.89 ± 0.17	0.94 ± 0.14

*C* control rats, *PS* rats fed a palm oil/sunflower seed oil mixture, *R* rats fed rapeseed oil, *RTC* rats fed rapeseed oil enriched with α-tocopherol and coenzyme Q10, *RTCC* rats fed RTC plus canolol, IκBα: nuclear factor of kappa light polypeptide gene enhancer in B-cells inhibitor, alpha normalized density (aU), *ICAM-1* intercellular adhesion molecule-1 mRNA expression, *VCAM-1* vascular cell adhesion protein-1 mRNA expression, *NOS3* nitric oxide synthase-3 mRNA expression. Averages of 12 animals per group

### Mitochondrial biogenesis

**Fig. 4:**
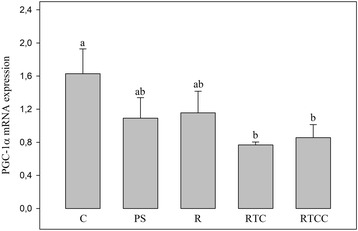
Mitochondrial biogenesis estimated as PGC-1α mRNA expression. Figures are averages of 12 rats per group. C: rats fed the control diet; PS: rats fed the high-fat diet rich in saturated and monounsaturated fatty acids; R: rats fed the high-fat diet rich in rapeseed oil; RTC: rats fed the same diet as R, but enriched with vitamin E and CoQ10; RTCC: rats fed the same diet as RTC, but enriched with canolol; PGC-1α: peroxisome proliferator activated receptor alpha; a,b: In a given panel, histograms without a common letter are significantly different

This pathway was evaluated by determining PGC-1α mRNA expression (Fig. 4). The various inter-group comparisons tested here (PS diet vs. C diet, R diet vs. PS diet, RTC and RTCC diets vs. R diet) did not find any significant differences.

## Discussion

The purpose of this study was to evaluate the effects of ALA-rich rapeseed oil on some aspects of cardiac activity in the context of a high-fat diet and to see whether the addition of different rapeseed-sourced antioxidants (vitamin E, CoQ10 and canolol) improved the effects of the refined rapeseed oil. The main significant results obtained during the study are summarized in Table 6.

**Table 6:** Summary of the main significant results

	PS vs. C	R vs. PS	RTC vs. R	RTCC vs. RTC
PL PUFA				
ω6 PUFAs	↓ 18:2 and ↑ 20:4, C22:4, C22:5;	↑ 18:2, ↓ 20:4, 22:4 and 22:5;	-	-
ω3 PUFAs	↓	↑ 18:3, 22:5 and 22:6	-	-
PUFAs/SFAs	↓	↑	-	-
Ag2 pathway				
mRNA Ag2R-1b	-	-	↑	↓
Collagen	-	-	↑	↓
Metabolism				
mRNA for PDK4	-	-	↑	↓
DG	↓	↑	-	-
TG	-	-	-	↓
Oxidative stress				
Thiol group	-	↑	-	-
TBARS	↓	-	-	-
FRAP	↓	-	-	-
SOD activity	↓	-	-	-
GPX activity	-	-	↑	-
mRNA for SOD2	-	-	↑	↓
mRNA for GPX4	-	-	↑	-
Apoptosis				
Cleaved caspase-3	↓	-	-	-
p53 mRNA	-	-	↓	-

*PS vs. C* comparison between rats fed the high-saturated fatty acid diet and rats fed the control diet, *R vs. PS* influence of rapeseed oil in the context of a high-fat diet, *RTC vs. R* influence of the combination of vitamin E and coenzyme Q10 in the context of a high-fat diet enriched with rapeseed oil, RTCC vs. *RTC* influence of canolol in the context of a high-fat diet enriched with rapeseed oil, vitamin E and coenzyme Q10, *PL* phospholipid, *PUFAs* polyunsaturated fatty acids, *SFAs* saturated fatty acids, *Ag2* angiotensin 2, *mRNA* messenger ribonucleic acid, *PDK4* pyruvate dehydrogenase kinase-4, *DG* diglyceride, *TG* triglyceride, *TBARS* thiobarbituric acid reactive substances, *FRAP* ferric reducing ability of plasma, *SOD* superoxide dismutase, *GPX* glutathione peroxidase

### Effects of the different diets on cardiac phospholipid fatty acid composition

The high-fat diet rich in saturated and monounsaturated fats (PS diet) triggered an increase in cardiac phospholipid SFA that was offset by a reduction in PUFA. These changes were associated with profound shifts in PUFA profile, probably in order to maintain membrane fluidity. The shortest and least unsaturated ω6 PUFA (LA and C20:2ω6) were drastically decreased and replaced by the longest and most unsaturated ω6 PUFAs (AA, C22:4ω6 and C22:5ω6). To compensate for this increased double bond index, all the ω3-series PUFA were reduced. The membrane lipid changes induced by the palm oil-rich PS diet are close to those provoked by a high-fat diet composed mainly of lard [5]. Interestingly, other data obtained in Zucker Diabetic Fatty rats indicated that the membrane lipid changes shown here were similar to those observed in hyperphagic animals fed a low-fat diet with compositionally-balanced PUFA [24]. The lipid profile of a classical high-fat diet thus appeared to have little effect on membrane fatty acid composition, which means the key inducer of the observed changes was likely the high calorie intake and/or amount of ingested fatty acids.

Our results showed that this conclusion does not apply to all high-fat diets. R diet, which is a source of ALA, led to a very different cardiac phospholipid fatty acid profile, as proportion of AA was normalized in comparison with the control group and C22:4 and C22:5ω6 were even reduced. These ω6 PUFAs were replaced by long-chain ω3 PUFAs (ALA, DPA and DHA). These modifications are classic responses when dietary ω6 PUFA are replaced by ω3 PUFA. They are due to ω6 and ω3 PUFA competing for incorporation in membrane phospholipids, and have already been seen with low-fat diets [11]. Supplementing the R diet with antioxidant did not modify membrane-phospholipid fatty acid composition.

### Effects of the different diets on oxidative stress

The different diets used here substantially modulated cardiac oxidative stress. The high-fat diets reduced oxidative stress compared to the control diet. This was characterized by a decrease in the amount of TBARS associated or not with an increase in thiol group preservation for the RSO-rich diets. These results can be explained by the PUFA profile of membrane phospholipids. The PS diet-induced increase in AA was already observed in one of our previous studies [5] evaluating the effects of a high-fat diet enriched with lard instead of palm oil. Coronary reactivity measurements indicated that the vasodilatation capacities of the microvessels were increased by one or more cyclooxygenase product(s). AA can have a strong effect on vessel dilatation via its cyclooxygenase products (prostaglandins such as prostacyclin). Thus, the high-fat diet-induced accumulation of AA probably increased coronary flow. The heart reacts like an engine: when it receives more fuel, i.e. more blood in this case, it contracts more intensively. Our previous work clearly indicated that the high-fat diet was associated with increased in vivo cardiac mechanical function [5]. It was not possible to measure cardiac mechanical function here. However, given the large increase in AA proportion of cardiac membranes, it is likely that myocardial contractility was increased. This data brings crucial insight to help understand the observations here. Indeed, the high-fat diets reduced oxidative stress despite a decrease in SOD activity. Obesity is known to increase oxidative stress [25]. However, our results clearly indicate an opposite effect that could at least occur in the heart during an early and short phase. To our knowledge, this is the first report of this kind of observations. The reduction of oxidative stress could be related to the likely increase in mechanical activity of the heart. Mechanical stimulation inevitably increases cardiomyocyte energy demand. As a result, the mitochondria increase their oxidative phosphorylation rate. This stimulation of oxidative phosphorylation reduces the mitochondrial ΔΨ: in isolated mitochondria, with ΔΨ being high during basal respiration and strongly reduced when oxidative phosphorylation is activated by ADP [26]. However, mitochondrial ROS release is high during basal respiration and strongly reduced by ADP addition [27]. A high ΔΨ is associated with numerous positive charges in the mitochondrial inter-membrane space which are able to extract electrons from the respiratory chain. These extracted electrons react with molecular oxygen to form superoxide anions. The relation between stimulation of mechanical function and reduced oxidative stress has already been observed in the heart in pathological situations such as the hyper-dynamic phase of sepsis [28]. Regular physical activity also reduces oxidative stress in skeletal muscle by decreasing the amount of TBARS [29] and enhancing GSH [30].

The reduced oxidative stress induced by the RSO-rich diets is more difficult to explain in relation to the PUFA profile of cardiac membranes, as proportions of AA were reduced to the levels observed in the control group. Therefore, the AA-induced activation of cardiac mechanical function was unable to fully explain the reduced oxidative stress induced by the RSO-rich diets. Cardiac mechanical performances were not determined here, so we do not know whether they were increased in the RSO-fed animals compared to controls, but such an increase would enable the animals to maintain their mobility despite excess fat and body masses. AA was normalized by dietary ALA-rich RSO, which suggests that the R diet did not increase coronary reactivity and mechanical function. However, several ω3 PUFA were increased. It is tempting to speculate that these fatty acids increased coronary micro-vessel dilatation, but there are only indirect evidences in the literature to support such an effect: ω3 fatty acid supplementation improves endothelial function and maximal oxygen uptake in endurance-trained athletes [31]; endothelial function is boosted by ω3 PUFA compared to saturated fats when circulating NEFA were increased by heparin treatment [32]; DHA-rich fish oil reverses the detrimental effects of SFA on postprandial vascular reactivity [33]. It is thus possible that dietary RSO improved coronary vessel dilatation and cardiac mechanical performances in this high-fat-diet context. The PS diets also decreased PUFA/SFA ratio while the ω3 PUFA-rich diets failed to restore this value to the level measured in the C group. The increased cardiac mechanical activity thought to occur with high-fat diets could result from this decreased PUFAs/SFAs ratio which may govern membrane fluidity and consequently cardiac function. ω3-PUFA are reported to increase membrane fluidity [34], which could perhaps sustain an increase in cardiac mechanical function as well as a decrease in mitochondrial ROS generation and cardiac oxidative stress.

### Effects of the different diets on cellular death

The reduced amounts of cleaved caspase-3 showed that the high-fat diets decreased the rate of apoptosis. This was true for the PS diets as well as for the R, RTC and RTCC diets. These results were probably related to oxidative stress, which produces cellular death through apoptosis and necrosis [35]. Thus, the high-fat-diet-induced reduction of oxidative stress led to downregulation of the apoptosis pathway in the present study. It also tended to reduce cardiac collagen content, since there was lower cell death and higher amounts of viable cardiomyocytes persisting in the myocardium. This was true for all the high-fat diets except RTC which maintained collagen content at a similar level to the control group. Fibrotic tissue was thus more abundant with the RTC diet than with the R and RTCC diets. The phenomenon was associated with increased angiotensin-2 receptor-1b (Ag2R-1b) mRNA expression.

The accumulation of adipose tissue in the obese state causes a rise in circulating angiotensin 2 [36]. In this context, adipose tissue contributes to at least 30% of plasma angiotensin. Ag2R-1a and -1b have potent vasoconstrictive effects on coronary vessels [37], and their activation by angiotensin 2 triggers autophagy and, in the long term, cardiac hypertrophy and heart failure [38]. Induced autophagy suggests cell death and fibrosis. The increased collagen content observed with the RTC diet can thus be explained by enhanced Ag2R-1b protein expression which potentiated the effects of angiotensin-2 on cellular death. The Ag2R activation-induced vasoconstriction of coronary micro-vessels probably triggered ischemia. Several features of myocardial activity suggested the occurrence of ischemia in this study. The RTC diet was associated with increased oxidative stress compared to the R diet, as signaled by an increase in GPx activity and SOD-2 and GPX-4 mRNA expression. However, ischemia is inevitably associated with increased ROS generation [39]. The observed changes evoked increased ROS production, which can induce cellular death as ω3-PUFA are vulnerable to ROS attack. The oxidative stress led to mitochondrial dysfunctions: the rise of myocardial triglyceride content suggested a decrease in β-oxidation rate. Furthermore, the increased pyruvate dehydrogenase kinase-4 mRNA expression evoked a simultaneous decrease in glucose oxidation. Reductions of glucose and fatty acid oxidation rates probably led to ATP deficiency. However, ischemia is well known to trigger ATP breakdown [40], and to provoke cellular death through necrosis and apoptosis [41, 42]. However, apoptosis occurs when there is still enough ATP present in the cardiomyocytes to activate the apoptosome, whereas necrosis occurs with ATP deficiency. Our data showed that the high rate of cellular death in the RTC group occurred mainly through necrosis. Here, the quantification of p53 mRNA expression helped estimate the type of cellular death occurring with this diet. p53 protein is anti-apoptotic: p53 mRNA expression was reduced by the RTC diet compared to the R diet, which suggested that the cardiomyocytes attempted to increase apoptosis, as necrosis was high due to the ATP deficiency. This was then confirmed by the caspase-3 activity, which tended to be reduced with the RTC diet compared to the R diet. The high rate of necrosis observed with the RTC diet suggests that the Ag2R-1b mRNA overexpression was efficient in triggering ischemia.

Supplementation of the RTC diet with canolol reversed the situation, preventing the overexpression of Ag2R-1b and occurrence of ischemia, as demonstrated by the reductions in fibrosis (collagen content restored to levels observed with the R diet), metabolic dysfunctions (re-activation of β-oxidation and glucose degradation rates) and oxidative stress (decrease in SOD2 mRNA expression).

The reason why vitamin E + CoQ10 increased Ag2R-1b mRNA expression in the context of a high-fat RSO-enriched diet remains unknown. The increase was probably related to the doses of vitamin E and CoQ10 chosen for this study. It has previously been shown that excess vitamin E is deleterious for isolated mitochondria [43], as it induces the opening of the mitochondrial permeability transition pore (mPTP) in the presence of calcium and thus promotes cell death. mPTP opening is provoked by excess oxidative stress [44]. High amounts of tocopheryl radicals in the mitochondrial membrane environment can thus trigger cellular death. It can also send signals to the nucleus to increase Ag2R-1b mRNA and protein expressions. The excess radical charge of tocopheryl compounds is usually detoxified by hydrophilic antioxidants such as vitamin C. In accordance with this hypothesis, addition of canolol to the RTC diet restored the situation observed with the R food. It is possible that canolol, despite being lipophilic, played the role of vitamin C and decreased the amount of tocopheryl radicals in the direct environment of the mitochondrial membrane. This process may be accompanied by a reduction in Ag2R-1b protein expression and in related toxicity.

### Limitations of the study

This study used single doses of vitamin E, CoQ10 and canolol. It is possible that lower doses of vitamin E and CoQ10 would not be noxious through induction of ischemia, in which case the protective effects of canolol would not have been observed. In contrast, lower doses of vitamin E and CoQ10 may have been able to exert a cardioprotective effect by acting as an antioxidant therapy. The study was not designed to answer these questions. Likewise, lower or higher doses of canolol could be either without effect or noxious. Further studies with varied doses of each of these antioxidants along with an ALA-rich diet may confirm/argue against the findings reported in the present study. Such studies are necessary before an adequate dietary formula can be determined.

## Conclusion

Our results indicated that the palm-oil-rich high-fat diet (PS diet) used here decreased cardiac oxidative stress, due to the increased accumulation of arachidonic acid in membrane phospholipids which, according to previous results collected in our laboratory, stimulates coronary perfusion and cardiac mechanical activity. As a result, energy metabolism was probably activated, which could reduce mitochondrial ΔΨ and ROS generation. These changes were logically associated with reduced apoptosis and fibrosis. The high-fat diet enriched with rapeseed oil (R diet) had similar effects on cardiomyocyte activity, although it normalized the AA proportion of cardiac phospholipids. However, rapeseed oil contains ALA, a ω3 fatty acid, which increased membrane accumulation of long-chain ω3 fatty acids such as ALA, DPA and DHA. The cyclooxygenase products derived from these fatty acids can have similar dilatation effects on coronary micro-circulation to the cyclooxygenase products formed from AA. When the R diet was enriched with vitamin E and CoQ10 (RTC diet), cardiac fibrosis was increased due to the occurrence of ischemia. The phenomenon was probably related to the accumulation of tocopheryl radicals in the environment of the mitochondrial membranes and an increased expression of angiotensin-2 receptor 1-b which has vasoconstriction properties. This ischemia was associated with deep mitochondrial dysfunctions characterized by a lower capacity to oxidize fatty acid and glucose and, finally, to synthesize ATP. Addition of canolol to the RTC diet suppressed the deleterious effects of excess vitamin E + CoQ10, probably by deactivating tocopheryl radicals in the mitochondria environment.

Taken together, our results indicated that ALA-rich rapeseed oil had an interesting effect since it strongly reduced the pro-inflammatory AA in cardiac phospholipids in favor of the anti-inflammatory long-chain ω3 PUFAs; without increasing oxidative stress: in the long term, dietary ALA could prevent the occurrence of cardiac failure due to obesity. However, we noted that the occurrence of a sustained oxidative stress (induced by dietary vitamin E + CoQ10 supplementation probably in excess in our experimental conditions) precipitated the cardiomyocytes toward ischemia, necrosis and fibrosis, which can accelerate the occurrence of cardiac failure. This effect could be due to the particular vulnerability of membrane ω3 fatty acids to ROS attack. Administration of an adequate antioxidant such as canolol in association with dietary ω3 PUFA thus appears essential in order to protect the organism during the pathophysiological situations involving sustained oxidative stress.

## Abbreviations

AA: Arachidonic acid
ADP: Adenosine diphosphate
Ag2R: Angiotensin-2 receptor
Akt: Protein kinase B
ALA: α-linolenic acid
ANOVA: Analysis of variance
ATP: Adenosine triphosphate
C: Control
cDNA: Complementary deoxyribonucleic acid
CoQ10: Coenzyme Q10
DG: Diglyceride
DHA: Docosahexaenoic acid
DPA: Docosapentaenoic acid
DTNB: Ellman’s reagent
ECL: Enhanced chemiluminescence
EDTA: Ethylenediaminetetraacetic acid
EPA: Eicosapentaenoic acid
Fe^III^-TPTZ: Ferric-tripyridyl triazine
FRAP: Ferric reducing ability of plasma
GAPDH: Glyceraldehyde-3-phosphate dehydrogenase
GPx: Glutathione peroxidase
GSH: Reduced glutathione
H_2_O_2_: Hydrogen peroxide
HEPES: 4-(2-hydroxyethyl)-1-piperazineethanesulfonic acid
HRP: Horseradish peroxidase
ICAM: Intercellular adhesion molecule
ITERG: Institut des Corps Gras
IκBα: Nuclear factor of kappa light polypeptide gene enhancer in B-cells inhibitor, alpha
LA: Linoleic acid
MMP9: matrix metallopeptidase 9
mRNA: messenger ribonucleic acid
NAC: N-acetyl-cysteine
NCSS: Number cruncher statistical system
NEFA: Nonesterified fatty acid
NoNo: Non-POU domain-containing octamer-binding protein
p: Probability
p53: Tumor protein p53
PDK: Pyruvate dehydrogenase kinase
PGC-1α: Peroxisome proliferator-activated receptor gamma coactivator 1-alpha
PS: Palm oil/sunflower oil
PUFA: Polyunsaturated fatty acid
PVDF: Polyvinylidene fluoride
qRT-PCR: Real-time quantitative reverse transcription polymerase chain reaction
R: Rapeseed oil
ROS: Reactive oxygen species
RTC: Rapeseed oil + α-tocopherol + coenzyme Q10
RTCC: Rapeseed oil + α-tocopherol + coenzyme Q10 + canolol
SDS: Sodium dodecyl sulfate
SEM: Standard error of the mean
SFA: Saturated fatty acid
SOD: Superoxide dismutase
TBARS: Thiobarbituric acid reactive substances
TG: Triglyceride
TGF-β1: Transforming growth factor beta 1
VCAM: Vascular cell adhesion molecule.
ΔΨ: Mitochondrial membrane potential
